# Supplementary material for: Common and Differential Traits of the Membrane Lipidome of Colon Cancer Cell Lines and Their Secreted Vesicles: Impact on Studies Using Cell Lines
Source: Cancers (Basel). 2020 May 20;12(5):1293. doi: 10.3390/cancers12051293 (PMC7281030; doi:10.3390/cancers12051293)
Supplement: Supplementary file 1 [file cancers-12-01293-s001.pdf]

cancers MDPI

Supplemental Materials: Common and Differential Traits of the Membrane Lipidome of Colon Cancer Cell Lines and their Secreted Vesicles: Impact on Studies Using Cell Lines

Table S1. Phospholipid and Sphingolipid lipid composition of commercial colon cell lines.

PL or SL Class	Prim		HT29		LS174t		SW480		Colo 201	
	Mean	±SD	Mean	±SD	Mean	±SD	Mean	±SD	Mean	±SD
**PC**	44.9	±4.8	50.4	±2.9[a]	49.2	±0.7	53.6	±1.6 [aaa]	53.6	±1.2 [aaa]
**SM**	11.1	±2.1	7.2	±1.0[aa]	9.0	±1.6	6.0	±0.6 [aaa,c]	7.8	±0.9[a]
**Cer**	1.4	±0.7	1.8	±0.5	1.2	± 0.2	1.5	±0.2	0.6	±0.0 [bbb,d]
**PE**	25.1	±3.4	8.2	±1.3[aaa]	11.7	±1.3 [aaa,bb]	10.8	±0.7 [aaa]	11.6	±1.0 [aaa,b]
**PE-P**	5.4	±0.6	18.5	±1.4[aaa]	14.8	±0.9 [aaa,bbb]	13.4	±1.1 [aaa,bbb]	13.9	±1.0 [aaa,bbb]
**PI**	4.8	±0.6	7.5	±1.1[aaa]	5.6	±0.8 [bb]	6.4	±0.6	5.4	±0.4 [bb]
**PS**	7.4	±0.2	6.4	±2.2	8.5	±0.8	8.4	±1.9	7.1	±0.9

Values are expressed as a percentage of total membrane lipid (mole %) and represent the mean ± SD, n = 3–6. Statistical significance was assessed using ANOVA followed by Bonferroni post-test analysis. a $p < 0.05$, aa $p < 0.01$, aaa $p < 0.001$, Primary vs HT29, LS174t, SW480 or Colo 201; b $p < 0.05$, bb $p < 0.01$, bbb $p < 0.001$, HT29 vs LS174t, SW480 or Colo 201; c $p < 0.05$, cc $p < 0.01$, ccc $p < 0.001$, LS174t vs SW480 or Colo 201; d $p < 0.05$, dd $p < 0.01$, ddd $p < 0.001$, SW480 vs Colo 201. Abbreviations: Cer: ceramide; PC: phosphatidylcholine; PE: phosphatidylethanolamine, PE-P: alkenyl phosphatidylethanolamine; PI: phosphatidylinositol; PL: phospholipid; PS: phosphatidylserine; SM: sphingomyelin; SL: sphingolipid. Numbers in bold are the mean values.

Table S2. Phospholipid and Sphingolipid molecular species composition of colon commercial cell lines.

Molecular Species	Prim		HT29		LS174t		SW480		Colo 201	
	Diacyl-phosphatidylcholine									
	Mean	±SD	Mean	±SD	Mean	±SD	Mean	±SD	Mean	±SD
32:0	**6.3**	±1.0	**9.5**	±1.8[aa]	**6.7**	±0.3[bb]	**3.8**	±0.7[a,bbb,c]	**5.3**	±0.4[bbb]
34:2	**13.1**	±3.4	**10.0**	±1.0[a]	**7.1**	±0.9[aaa,b]	**6.8**	±0.3 [aaa, b]	**7.9**	±0.5[aaa]
34:1	**34.7**	±0.8	**39.1**	±2.8[a]	**43.6**	±1.9[aaa,bb]	**51.0**	±1.2[aaa,bb,ccc]	**42.1**	±1.4[aaa,ddd]
36:4	**1.5**	±0.1	**2.8**	±1.2	**1.9**	±0.6	**2.3**	±0.2	**0.7**	±0.1 [bbb,c,d]
36:3	**5.5**	±1.0	**4.2**	±0.6[aa]	**3.5**	±0.3[aaa]	**2.9**	±0.2[aaa,bb]	**2.7**	±0.1[aaa,bbb,c]
36:2	**21.8**	±1.7	**16.6**	±2.8[a]	**17.0**	±2.0	**13.9**	±1.2[aa]	**27.3**	±3.1[a,bbb,ccc,ddd]
36:1	**7.4**	±0.4	**7.1**	±0.7	**8.7**	±1.1	**9.2**	±0.8	**9.2**	±2.2
38:6	**0.1**	±0.0	**1.8**	±0.6[aaa]	**1.3**	±0.3[aa]	**1.5**	±0.1[aaa]	**0.5**	±0.1[bbb,c,dd]
38:5	**1.0**	±0.2	**2.7**	±1.1[a]	**2.2**	±0.5	**2.7**	±0.3[a]	**1.0**	±0.2[bb,dd]
38:4	**4.1**	±0.6	**2.3**	±1.3[a]	**2.8**	±0.5	**2.1**	±0.2[a]	**0.7**	±0.1[aaa,b,cc]
38:3	**3.0**	±0.4	**1.5**	±0.5[aaa]	**2.4**	±0.4[bb]	**1.4**	±0.2[aaa,c]	**1.1**	±0.2 [aaa,ccc]
40:7	**0.1**	±0.0	**0.6**	±0.1[aaa]	**0.5**	±0.1 [aaa]	**0.5**	±0.0[aaa]	**0.3**	±0.0[aaa,bbb,d]
40:6	**0.2**	±0.0	**1.0**	±0.5[a]	**0.9**	±0.3[a]	**0.8**	±0.1	**0.4**	±0.2[b]
40:5	**0.5**	±0.0	**0.9**	±0.4	**1.0**	±0.3	**1.0**	±0.1	**0.6**	±0.1
40:4	**0.8**	±0.1	**0.2**	±0.1[aaa]	**0.3**	±0.1[aaa]	**0.2**	±0.1[aaa]	**0.2**	±0.0[aaa,c]
	Diacyl-phosphatidylethanolamine									
32:0	**0.3**	±0.2	**0.5**	±0.2	**0.5**	±0.2	**0.3**	±0.0	**0.3**	±0.1
34:2	**4.2**	±1.1	**4.1**	±1.0	**2.5**	±0.6[a,bb]	**1.9**	±0.3[aa,bbb]	**2.7**	±0.3[b]
34:1	**13.0**	±1.8	**16.0**	±1.7	**14.8**	±1.6	**13.0**	±1.2[b]	**14.8**	±0.8
36:4	**0.7**	±0.1	**2.0**	±0.3[aaa]	**0.8**	±0.1[bbb]	**1.0**	±0.2[bbb]	**0.2**	±0.1[bbb,cc,ddd]
36:3	**4.9**	±1.5	**4.8**	±0.9	**2.1**	±0.6[aaa,bbb]	**3.2**	±0.5[b]	**3.0**	±0.2[a,bb]
36:2	**34.4**	±5.3	**19.9**	±3.8[aa]	**19.7**	±4.5[aaa]	**17.9**	±0.8[aaa]	**32.1**	±5.4[bb,cc,ddd]
36:1	**14.1**	±2.2	**9.9**	±2.0	**18.0**	±1.1[bbb]	**16.7**	±1.7[bbb]	**25.5**	±3.4[aaa,bbb,ccc,ddd]
38:5	**3.1**	±0.3	**15.1**	±2.9[aaa]	**6.6**	±1.3[bbb]	**10.1**	±1.9[aaa,bb]	**3.7**	±0.7[bbb,ddd]

38:4	**8.9**	±0.5	**12.7**	±1.7[aa]	**12.5**	±1.3[a]	**14.3**	±1.8[aaa]	**4.7**	±1.0[aa,bbb,ccc,ddd]
38:3	**10.6**	±0.9	**3.7**	±1.1[aaa]	**6.3**	±2.0[aa,b]	**5.3**	±0.6[aaa]	**2.5**	±0.5[aaa,ccc,d]
40:7	**0.3**	±0.1	**3.0**	±0.1[aaa]	**2.9**	±0.7[aaa]	**3.0**	±0.1[aaa]	**2.7**	±0.4[aaa]
40:6	**0.5**	±0.1	**3.5**	±0.9[aa]	**6.2**	±1.7[aaa,bb]	**4.8**	±0.7[aaa]	**4.1**	±0.6[aa,c]
40:5	**1.3**	±0.3	**1.4**	±0.4	**4.4**	±1.4[aaa,bbb]	**4.2**	±0.5[aa,bbb]	**2.7**	±0.5[c]
40:4	**3.4**	±1.6	**0.2**	±0.2[aaa]	**1.0**	±0.5[aaa]	**1.4**	±0.3[aa,b]	**0.3**	±0.1[aaa]
**Alkenyl-phosphatidylethanolamine**										
16:0/18:1	**11.7**	±1.0	**4.7**	±1.9[aaa]	**7.6**	±0.3[aa]	**6.1**	±0.6[aa]	**13.6**	±1.3[bb, d]
16:0/20:4	**14.4**	±2.8	**30.7**	±5.5[aaa]	**18.2**	±2.6[bbb]	**33.8**	±2.9[aaa, ccc]	**13.1**	±0.5[bbb, ddd]
18:1/18:1	**16.9**	±6.3	**1.3**	±0.4[aaa]	**2.6**	±0.6[aaa]	**1.2**	±0.3[aaa]	**7.7**	±0.6[aaa, bbb, cc, dd]
18:0/18:1	**18.1**	±2.4	**5.1**	±1.8[aaa]	**11.1**	±1.9[aaa, bbb]	**2.3**	±0.5[aaa, ccc]	**13.9**	±1.6[a, bbb, ccc]
16:0/22:6	**0.8**	±0.2	**19.2**	±4.4[aaa]	**19.4**	±3.5[aaa]	**26.9**	±2.7[aaa, b, c]	**15.8**	±2.4[aaa, ddd]
18:1/20:4	**16.9**	±1.6	**3.7**	±1.7[aaa]	**3.0**	±1.1[aaa]	**4.8**	±0.3[aaa]	**4.8**	±0.5[aaa]
18:0/20:4	**21.3**	±0.8	**21.7**	±4.5	**18.8**	±3.5	**13.7**	±0.7[bb]	**13.3**	±1.4[a, bb]
18:1/22:6	**0.4**	±0.1	**1.1**	±0.4	**1.7**	±0.7	**1.9**	±0.4	**3.5**	±0.8[aaa, bbb, ccc, dd]
18:0/22:6	**0.8**	±0.0	**12.5**	±2.6[aaa]	**17.6**	±2.1[aaa, bb]	**9.4**	±1.9[aaa, ccc]	**14.2**	±1.1[aaa, d]
**Phosphatidylinositol**										
34:2	**0.7**	±0.4	**1.3**	±0.3	**1.7**	±0.3[a]	**1.6**	±0.5[a]	**1.9**	±0.3[aa]
34:1	**0.9**	±0.8	**14.6**	±4.9[aaa]	**15.6**	±2.2[aaa]	**9.8**	±1.2[aa,c]	**5.1**	±0.4[bbb,ccc]
36:4	**0.6**	±0.1	**1.8**	±0.5[a]	**1.8**	±0.9[a]	**2.5**	±0.0[aa]	**1.2**	±0.1 [d]
36:3	**4.1**	±1.2	**2.8**	±0.4	**4.2**	±0.9[b]	**2.1**	±0.4[aa,ccc]	**3.0**	±0.4
36:2	**7.9**	±3.4	**7.5**	±1.3	**11.2**	±1.5[b]	**9.9**	±1.4	**16.2**	±1.8[aaa,bbb,cc,ddd]
36:1	**1.5**	±1.5	**23.6**	±7.0[aaa]	**17.4**	±2.0[aaa]	**16.2**	±1.7[aaa]	**9.6**	±0.9[bbb,c]
38:6	**0.1**	±0.0	**0.7**	±0.2[aa]	**0.6**	±0.4[a]	**0.6**	±0.0 [a]	**0.2**	±0.1[b]
38:5	**3.9**	±2.1	**2.9**	±0.7	**2.7**	±1.1	**4.1**	±0.3	**4.3**	±0.4
38:4	**27.8**	±9.7	**20.1**	±9.5	**15.9**	±5.4	**30.7**	±3.0[c]	**27.5**	±1.8
38:3	**47.9**	±4.8	**17.8**	±3.7[aaa]	**22.4**	±7.7[aaa]	**13.2**	±1.0[aaa,c]	**23.3**	±1.3[aaa,d]
40:7	**0.2**	±0.0	**0.3**	±0.1	**0.2**	±0.1	**0.5**	±0.1[a,cc]	**0.4**	±0.1[a,cc]
40:6	**0.5**	±0.1	**3.1**	±0.6[aaa]	**2.4**	±1.2[aa]	**2.1**	±0.2 [a]	**2.3**	±0.1[aa]
40:5	**1.3**	±0.1	**2.8**	±0.6[a]	**2.8**	±1.1[a]	**4.5**	±0.3[aaa,bb,cc]	**3.2**	±0.3[aa]

40:4	**2.6**	±0.1	**0.7**	±0.4[aaa]	**1.2**	±0.2[aaa]	**2.1**	±0.5[bbb,cc]	**1.6**	±0.3[aa,bb]
					**Phosphatidylserine**					
32:0	**0.1**	±0.1	**0.1**	±0.0	**0.1**	±0.0	**0.0**	±0.0[a]	**0.0**	±0.0[a]
34:2	**1.9**	±0.6	**2.1**	±0.5	**0.8**	±0.3[aa,bbb]	**0.4**	±0.1[aaa,bbb]	**0.7**	±0.2[aa,bbb]
34:1	**10.0**	±1.9	**16.7**	±2.3[aaa]	**7.6**	±1.4[bbb]	**6.9**	±0.6[bbb]	**7.8**	±0.6[bbb]
36:4	**0.2**	±0.0	**0.6**	±0.1[aaa]	**0.1**	±0.0[aaa,bbb]	**0.1**	±0.0[aa,bbb]	**0.0**	±0.0[aaa,bbb]
36:3	**1.5**	±0.2	**2.9**	±0.1[aaa]	**0.4**	±0.2[aaa,bbb]	**0.4**	±0.2[aaa,bbb]	**0.4**	±0.1[aaa,bbb]
36:2	**15.3**	±0.6	**16.4**	±1.8	**7.8**	±1.9[aaa,bbb]	**9.7**	±0.8[aa,bbb]	**11.1**	±1.7[a,bbb,c]
36:1	**53.7**	±2.0	**38.0**	±2.2[aaa]	**54.2**	±2.3[bbb]	**51.0**	±2.3[bbb]	**60.5**	±0.4[aa,bbb,ccc,ddd]
38:6	**0.0**	±0.0	**0.2**	±0.2	**0.1**	±0.1	**0.2**	±0.0	**0.0**	±0.0[b]
38:5	**0.4**	±0.0	**2.5**	±1.0[aaa]	**0.4**	±0.1[bbb]	**0.8**	±0.2[bbb]	**0.2**	±0.1[bbb]
38:4	**3.2**	±0.1	**6.0**	±2.1[a]	**2.6**	±0.4[bbb]	**3.2**	±0.7[bb]	**1.4**	±0.1[bbb]
38:3	**9.9**	±1.1	**6.8**	±1.1	**8.2**	±1.1	**6.6**	±1.0[aa]	**3.7**	±0.5[aaa,bbb,ccc,dd]
40:7	**0.0**	±0.0	**0.2**	±0.1[a]	**0.2**	±0.0[aa]	**0.4**	±0.1[aaa,b,c]	**0.4**	±0.0[aaa,b]
40:6	**0.2**	±0.1	**4.8**	±1.1[aaa]	**8.5**	±2.2[aaa,bbb]	**9.5**	±0.4[aaa,bbb]	**7.1**	±0.8[aaa]
40:5	**0.7**	±0.2	**2.4**	±0.6	**7.3**	±1.4[aaa,bbb]	**8.4**	±0.5[aaa,bbb]	**6.1**	±0.8[aaa,bbb,dd]
**Ceramides**										
d18:1/16:0	**36.8**	±3.3	**51.3**	±4.7[a]	**51.2**	±9.9[a]	**53.2**	±3.0[a]	**58.2**	±3.8[aa]
d18:0/16:0	**2.5**	±0.1	**9.1**	±2.5	**16.5**	±5.4[aaa,bb]	**6.2**	±2.6[ccc]	**2.2**	±0.2[b,ccc]
d18:1/18:1	**0.6**	±0.2	**1.2**	±0.5[a]	**0.7**[b]	±0.2	**1.6**	±0.1[aa,cc]	**1.0**	±0.2
d18:1/18:0	**3.3**	±0.7	**4.6**	±2.0	**2.7**	±0.9	**5.9**	±0.7[cc]	**3.7**	±0.3
d18:1/20:0	**1.1**	±0.0	**2.8**	±0.8[aa]	**1.8**	±0.7	**1.9**	±0.2	**2.6**	±0.4[a]
d18:1/22:1	**2.7**	±1.0	**2.9**	±0.8	**2.0**	±0.8	**2.7**	±0.4	**2.0**	±0.5
d18:1/22:0	**6.8**	±0.5	**5.7**	±1.2	**4.6**	±1.1[a]	**4.7**	±0.7	**4.8**	±1.2
d18:1/24:1	**36.5**	±6.8	**9.5**	±2.5[aaa]	**9.7**	±2.1[aaa]	**12.4**	±1.0[aaa]	**13.6**	±1.5[aaa]
d18:1/24:0	**7.0**	±0.8	**7.7**	±0.9	**6.0**	±1.2	**5.4**	±0.7[b]	**6.8**	±1.4
d18:1/26:1	**0.9**	±0.1	**0.3**	±0.0[aaa]	**0.4**	±0.1[aaa]	**0.2**	±0.1[aaa]	**0.3**	±0.0[aaa]
d18:1/26:0	**0.2**	±0.1	**0.3**	±0.1	**0.3**	±0.1	**0.1**	±0.0[bb,cc]	**0.2**	±0.1
d16:1/18:1	**1.7**	±0.6	**4.7**	±0.7[aa]	**4.0**	±1.0[a]	**5.8**	±0.4[aaa,c]	**4.6**	±1.2[aa]
**Sphingomyelin**										

d18:1/16:0	**38.2**	±3.3	**53.4**	±5.4	**56.2**	±7.9[aaa]	**38.2**	±2.5[bb,ccc]	**30.3**	±5.7[bbb,ccc]
d18:1/18:0	**1.6**	±0.1	**2.7**	±0.9	**1.1**	±0.5[bb]	**3.1**	±0.4[a,ccc]	**0.8**	±0.3[bbb,ddd]
d18:1/20:0	**0.6**	±0.0	**1.7**	±2.7	**0.4**	±0.2	**0.3**	±0.1	**0.2**	±0.1
d18:1/22:1	**0.5**	±0.0	**0.2**	±0.1	**0.3**	±0.1	**0.1**	±0.0 [a]	**0.2**	±0.3
d18:1/22:0	**6.2**	±0.6	**6.3**	±1.3	**5.9**	±1.9	**6.8**	±1.0	**4.6**	±1.4
d18:1/24:1	**26.7**	±1.8	**10.9**	±1.8[aaa]	**14.6**	±3.2[aaa]	**24.7**	±3.9[bbb,cc]	**23.5**	±4.8[bbb,cc]
d18:1/24:0	**24.8**	±3.2	**24.3**	±4.0	**20.3**	±7.1	**25.9**	±2.2	**37.2**	±7.4[a,bb,ccc,d]
d18:1/26:1	**0.8**	±0.1	**0.2**	±0.0[aa]	**0.2**	±0.2 [aa]	**0.2**	±0.1[aa]	**0.8**	±0.3[bbb,cc,dd]
d18:1/26:0	**0.5**	±0.1	**0.7**	±0.6	**1.4**	±1.1	**0.8**	±0.6	**2.5**	±1.4[b]

Comprehensive lipidome analysis of Primary, HT29, LS174t, SW4380 and Colo 201 cells. Statistical significance was assessed using ANOVA followed by Bonferroni post-test analysis. a $p < 0.05$, aa $p < 0.01$, aaa $p < 0.001$, Primary vs HT29, LS174t, SW480 or Colo 201; b $p < 0.05$, bb $p < 0.01$, bbb $p < 0.001$, HT29 vs LS174t, SW480 or Colo 201; c $P < 0.05$, cc $p < 0.01$, ccc $p < 0.001$, LS174t vs SW480 or Colo 201; d $p < 0.05$, dd $p < 0.01$, ddd $p < 0.001$, SW480 vs. Colo 201. Abbreviations: Cer: ceramide; PC: phosphatidylcholine; PE: phosphatidylethanolamine, PE-P: alkenyl phosphatidylethanolamine; PI: phosphatidylinositol; PS: phosphatidylserine; SM: sphingomyelin. Numbers in bold are the mean values.

Table S3. Distribution of the lipid molecular species within the main membrane lipids of colon commercial cell lines.

Molecular Species	Prim		HT29		LS174t		SW480		Colo201	
	Mean	±SD	Mean	±SD	Mean	±SD	Mean	±SD	Mean	±SD
					**1st Group**					
PC38:4	**28.7**	±5.9	**13.8**	±6.5	**20.6**	±4.0	**17.0**	±1.3[a]	**9.1**	±1.6[aaa,cc]
PE38:4	**35.1**	±2.4	**13.3**	±3.4[aaa]	**21.9**	±1.6[aaa,bbb]	**23.0**	±3.3[aaa,bbb]	**12.6**	±3.1[aaa,ccc,ddd]
PEP38:4	**12.5**	±7.0	**50.6**	±7.8[aaa]	**41.3**	±3.4[aaa]	**27.3**	±3.3[a,bbb,cc]	**42.0**	±3.8[aaa,dd]
PI38:4	**19.9**	±3.2	**17.9**	±3.3	**12.8**	±2.4[a]	**28.8**	±1.5[aa,bbb,ccc]	**34.2**	±2.8a[aa,bbb,ccc]
PS38:4	**3.8**	±1.1	**4.5**	±1.0	**3.3**	±0.3	**3.9**	±1.0	**2.2**	±0.4[bbb,d]
PC36:4	**43.5**	±9.2	**18.4**	±5.2	**24.2**	±4.1[aaa]	**20.1**	±1.2[aaa]	**15.7**	±2.4[aaa]
PE36:4	**10.4**	±0.6	**2.3**	±0.5[aaa]	**2.6**	±0.7[aaa]	**1.9**	±0.5[aaa]	**1.2**	±0.8[aaa,c]
PEP36:4	**43.5**	±9.8	**77.0**	±4.8[aaa]	**70.5**	±5.7[aaa]	**75.2**	±1.9[aaa]	**80.2**	±2.0[aaa]
PI36:4	**1.8**	±0.4	**1.8**	±0.4	**2.6**	±1.5	**2.7**	±0.5	**2.9**	±0.3
PS36:4	**0.8**	±0.1	**0.6**	±0.3	**0.1**	±0.0[aaa,bbb]	**0.1**	±0.0[aaa,bbb]	**0.0**	±0.0[aaa,bbb]
PC38:6	**37.2**	±10.5	**20.0**	±10.6	**17.0**	±4.9[aa]	**17.1**	±0.9[a]	**11.1**	±3.0[aaa]
PE38:6	**32.1**	±5.3	**5.4**	±0.6[aaa]	**5.2**	±2.6[aaa]	**6.5**	±1.5[aaa]	**3.2**	±0.5[aaa]
PEP38:6	**27.6**	±8.1	**73.3**	±10.5[aaa]	**76.6**	±7.9[aaa]	**75.2**	±2.1[aaa]	**85.1**	±3.2[aaa]
PI38:6	**2.2**	±1.2	**1.0**[a]	±0.1	**0.9**	±0.6	**0.8**	±0.1[a]	**0.5**	±0.2[aa]
PS38:6	**1.0**	±0.9	**0.3**	±0.2	**0.4**	±0.2	**0.4**	±0.1	**0.1**	±0.1 [a]
PC40:6	**31.2**	±10.5	**14.2**	±9.1	**9.6**	±3.4[aa]	**13.1**	±2.9[a]	**6.1**	±3.7[aaa]
PE40:6	**45.6**	±5.3	**8.2**	±3.4[aaa]	**15.6**	±3.5[aaa,b]	**16.9**	±2.1[aaa,bb]	**14.5**	±3.1[aaa]
PEP40:6	**9.6**	±8.4	**62.9**	±10.3[aaa]	**56.7**	± 5.4[aaa]	**40.2**	±2.0[aaa,bbb,c]	**60.1**	±5.4[aaa,dd]
PI40:6	**7.7**	±2.8	**6.4**	±1.6	**2.7**	±0.8[aaa,bbb]	**4.4**	±0.8[a]	**3.9**	±0.4[aa]
PS40:6	**5.9**	±1.1	**8.2**	±3.0	**15.4**	±2.4[aaa,bbb]	**25.4**	±3.4[aaa,bbb,ccc]	**15.4**	±1.5[aaa,bb,ddd]
					**2nd Group**					
PC38:5	**21.3**	±4.6	**36.5**	±7.6	**43.7**	±4.3[aaa]	**41.2**	±4.2[aaa]	**28.9**	±3.9[cc,d]
PE38:5	**37.4**	±8.4	**34.7**	±5.3	**31.1**	±4.5	**31.1**	±4.9	**22.5**	±1.2[aa,bb]
PEP38:5	**32.0**	±10.7	**18.4**	±3.6[aa]	**17.5**	±4.1[aa]	**18.4**	±2.9[a]	**35.5**	±3.5[bbb,ccc,ddd]
PI38:5	**8.0**	±3.1	**6.3**	±1.6	**6.3**	±2.8	**7.5**	±0.9	**12.4**	±1.2[bbb,ccc,d]
PS38:5	**1.4**	±0.4	**4.2**	±1.1[aaa]	**1.4**	±0.4[bbb]	**1.8**	±0.6[bbb]	**0.7**	±0.3[bbb]
PC40:7	**22.3**	±3.0	**37.5**	±8.8	**26.8**	±3.0[b]	**29.8**	±1.9	**18.0**	±2.6[bbb,d]
PE40:7	**53.6**	±14.1	**31.5**	±4.2[aa]	**40.0**	±10.5	**35.0**	±1.8	**30.6**	±2.8[aa]
PEP40:7	**15.0**	±11.6	**26.5**	±8.7[aaa]	**29.5**	±11.7	**28.2**	±2.2	**46.5**	±5.3[b,c]
PI40:7	**7.6**	±1.6	**2.7**	±1.2[aaa]	**1.2**	±0.5[aaa]	**3.2**	±0.2[aaa,c]	**2.2**	±0.7[aaa]
PS40:7	**1.5**	±0.9	**1.8**	±1.5	**2.4**	±0.4	**3.8**	±1.3	**2.7**	±0.3
					**3rd Group**					
PC32:0	**96.7**	±0.9	**99.0**	±0.5	**98.2**	±0.7[a]	**98.5**	±0.2[aa]	**98.9**	±0.4[aaa]
PE32:0	**3.0**	±1.1	**0.8**	±0.5[aaa]	**1.6**	±0.5[a]	**1.4**	±0.2[a]	**1.0**	±0.4[aaa]
PS32:0	**0.3**	±0.1	**0.1**	±0.1	**0.2**	±0.1	**0.2**	±0.1	**0.1**	±0.1 [a]
PC34:2	**82.5**	±2.9	**89.9**	±2.7	**88.7**	±1.5[aa]	**91.4**	±1.6[aaa]	**90.2**	±0.9[aaa]
PE34:2	**15.1**	±3.0	**5.9**	±1.5[aaa]	**7.3**	±0.8[aaa]	**5.1**	±0.9[aaa]	**6.6**	±0.8[aaa]
PI34:2	**0.5**	±0.1	**1.7**	±0.4 [a]	**2.4**	±0.4[aaa]	**2.6**	±0.9[aaa]	**2.2**	±0.2[aaa]
PS34:2	**1.9**	±0.0	**2.4**	±1.0	**1.6**	±0.6	**0.8**	±0.3 [b]	**1.0**	±0.2 [b]
PC34:1	**75.9**	±0.1	**82.0**	±4.2	**83.1**	±0.9[aa,c]	**88.8**	±0.9[aaa,bb]	**83.6**	±0.9[aa,d]
PE34:1	**16.0**	±2.1	**5.4**	±0.5[aaa]	**6.6**	±0.4[aaa]	**4.6**	±0.6[aaa,cc]	**6.3**	±0.3[aaa]
PEP34:1	**4.4**	±1.6	**3.6**	±1.3	**4.4**	±0.3	**2.6**	±0.4	**7.0**	± 1.0[a,bbb,cc,ddd]
PI34:1	**0.2**	±0.2	**4.6**	±1.5[aaa]	**3.4**	±0.7[aaa]	**2.0**	±0.4[bb]	**1.0**	±0.1[bbb,cc]
PS34:1	**3.6**	±0.4	**4.5**	±1.6	**2.5**	±0.5[b]	**1.9**	±0.5[bb]	**2.1**	±0.3[bb]

PC36:3	**61.9**	±6.0	**72.4**	±6.7	**77.2**	±4.0^{aa}	**75.3**	±2.3^{a}	**72.5**	±2.7
PE36:3	**30.2**	±4.0	**13.7**	±4.0^{aaa}	**10.9**	±3.3^{aaa}	**16.6**	±1.6^{aaa}	**17.7**	±2.2^{aaa,c}
PI36:3	**5.0**	±1.7	**7.3**	±1.7	**10.5**	±2.7^{aa}	**6.4**	±1.1^{c}	**8.2**	±1.3
PS36:3	**2.8**	±0.4	**6.6**	±2.8^{a}	**1.4**	±0.9^{bbb}	**1.7**	±1.3^{bb}	**1.6**	±0.3^{bbb}
PC36:2	**46.4**	±4.9	**70.6**	±4.0	**68.1**	±1.2^{aaa}	**67.7**	±1.8^{aaa}	**69.4**	±2.2^{aaa}
PE36:2	**41.1**	±8.3	**13.8**	±3.4^{aaa}	**18.3**	±1.5^{aaa}	**17.7**	±2.1^{aaa}	**17.6**	±2.6^{aaa}
PEP36:2	**5.2**	±1.6	**2.0**	±0.6^{aaa}	**3.2**	±0.7^{a}	**1.4**	±0.1^{aaa,c}	**5.1**	± 0.7^{bbb,cc,ddd}
PI36:2	**1.9**	±1.1	**4.7**	±0.7^{aaa}	**5.1**	±1.0^{aaa}	**5.7**	±0.8^{aaa}	**4.2**	±0.4^{a}
PS36:2	**5.4**	±0.7	**8.8**	±2.5	**5.3**	±0.9^{bb}	**7.4**	±1.7	**3.7**	±0.4^{bbb,d}
					**4th Group**					
PC36:1	**28.1**	±3.5	**38.2**	±8.4	**31.4**	±3.1	**40.1**	±5.2	**33.4**	±3.6
PE36:1	**29.9**	±6.6	**8.7**	±2.9^{aaa}	**15.5**	±1.8^{aaa,bb}	**14.7**	±1.5^{aaa,b}	**20.1**	±1.4^{aa,bbb}
PEP36:1	**8.1**	±0.9	**9.7**	±2.5	**12.1**	±2.0^{a}	**2.5**	±0.7^{aa,bbb,ccc}	**13.2**	±0.9^{aa,b,ddd}
PI36:1	**0.7**	±0.8	**18.5**	±5.1^{aaa}	**7.2**	±1.5^{a,bbb}	**8.4**	±1.6^{a,bbb}	**3.7**	±0.8^{bbb}
PS36:1	**33.3**	±1.7	**24.9**	±5.4	**33.8**	±2.5^{b}	**34.4**	±6.1^{b}	**29.6**	±3.7
PC38:3	**19.1**	±3.4	**26.2**	±6.1	**30.3**	±2.3^{aa}	**28.1**	±1.9	**24.3**	±2.8
PE38:3	**37.9**	±5.8	**10.9**	±3.9^{aaa}	**19.1**	±4.7^{aaa,b}	**21.1**	±1.4^{aaa,bb}	**12.0**	±1.6^{aaa,d}
PI38:3	**32.5**	±4.3	**47.2**	±5.6^{a}	**31.9**	±8.6^{bb}	**30.9**	±1.1^{bb}	**52.8**	±3.7^{aa,ccc,ddd}
PS38:3	**10.5**	±1.7	**15.8**	±6.6	**18.7**	±4.0	**20.0**	±2.6	**10.9**	±1.8^{d}
PC40:5	**32.0**	±3.1	**45.5**	±14.7	**27.6**	±8.8^{b}	**26.5**	±3.5^{b}	**25.8**	±3.7^{b}
PE40:5	**50.2**	±1.8	**13.2**	±4.7^{aaa}	**28.8**	±5.6^{aaa,bbb}	**23.3**	±3.6^{aaa,b}	**25.1**	±1.3^{aaa,bb}
PI40:5	**9.6**	±2.1	**23.9**	±7.6^{aa}	**8.5**	±2.2^{bbb}	**14.6**	±1.5^{b}	**14.2**	±2.1^{b}
PS40:5	**8.3**	±1.9	**17.4**	±7.7	**35.1**	±2.2^{aaa,bbb}	**35.6**	±5.9^{aaa,bbb}	**34.9**	±4.7^{aaa,bbb}
PC40:4	**23.4**	±1.2	**49.9**	±16.9	**33.6**	±10.3^{bb}	**20.3**	±9.0	**34.3**	±6.7
PE40:4	**54.0**	±3.3	**8.8**	±6.2^{aaa}	**24.3**	±10.2^{aaa,bb}	**24.9**	±5.4^{aaa,b}	**13.6**	±4.1^{aaa}
PI40:4	**8.7**	±2.0	**29.6**	±18.0	**14.5**	±5.5	**21.0**	±5.1	**34.7**	±7.8^{a,c}
PS40:4	**13.9**	±2.0	**11.7**	±8.4	**27.6**	±3.6^{bb}	**33.7**	±10.7^{a,bbb}	**17.4**	±4.6^{d}

Statistical significance was assessed using ANOVA followed by Bonferroni post-test analysis. a $p < 0.05$, aa $p < 0.01$, aaa $p < 0.001$, Primary vs HT29, LS174t, SW480 or Colo 201; b $p < 0.05$, bb $p < 0.01$, bbb $p < 0.001$, HT29 vs LS174t, SW480 or Colo 201; c $p < 0.05$, cc $p < 0.01$, ccc $p < 0.001$, LS174t vs SW480 or Colo 201; d $p < 0.05$, dd $p < 0.01$, ddd $p < 0.001$, SW480 vs. Colo 201. Abbreviations: PC: phosphatidylcholine; PE: phosphatidylethanolamine, PE-P: alkenyl phosphatidylethanolamine; PI: phosphatidylinositol; PS: phosphatidylserine. Numbers in bold are the mean values.

Table S4. Densitometry readings/intensity ratio of each band, for each WB included in this study.

			**FAR1 48 kDa**			
	**Sample**	**FAR1**	**β-actin**	**FAR1/β-actin**	**Normalization to primary cells**	**Primary cells FAR1/β-actin ratio**
Memb 1	Prim n=1	9.11	105.71	0.09	0.88	0.10
	Prim n=2	10.62	99.89	0.11	1.08	
Memb 3	Prim n=1	16.62	147.62	0.11	1.14	
	Prim n=2	18.99	149.98	0.13	1.29	
	Prim n=3	18.32	142.34	0.13	1.31	
Memb 4	Prim n=1	4.21	141.47	0.03	0.30	
Memb 1	Colo201 n=1	36.50	53.65	0.68	6.91	
	Colo201 n=2	40.23	58.05	0.69	7.04	
Memb 3	Colo201 n=1	77.51	98.31	0.79	8.01	
	Colo201 n=2	71.08	86.12	0.83	8.39	
	Colo201 n=3	84.11	72.36	1.16	11.82	
Memb 1	Ht29 n=1	75.75	71.01	1.07	10.84	
	Ht29 n=2	83.67	94.46	0.89	9.00	
Memb 3	Ht29 n=1	126.12	87.68	1.44	14.62	
	Ht29 n=2	144.85	103.43	1.40	14.24	
	Ht29 n=3	131.79	102.49	1.29	13.07	
Memb 1	LS174t n=1	28.79	62.34	0.46	4.69	
	LS174t n=2	22.89	72.57	0.32	3.21	
Memb 3	LS174t n=1	53.59	105.05	0.51	5.19	
	LS174t n=2	56.52	97.16	0.58	5.91	
	LS174t n=3	51.73	101.06	0.51	5.20	
Memb 1	SW480 n=1	65.98	66.50	0.99	10.09	
	SW480 n=2	64.80	89.44	0.72	7.37	
Memb 4	SW480 n=1	43.11	67.00	0.64	6.54	
	SW480 n=2	57.85	78.84	0.73	7.46	
	SW480 n=3	79.08	75.18	1.05	10.69	
			**AGPS 70 kDa**			
	**Sample**	**AGPS**	**β- actin**	**AGPS/β- actin**	**Normalization to primary cells**	**Primary cells AGPS/β-actin ratio**
Memb 1	Prim n=1	6.70	105.71	0.06	2.28	0.03

	Prim n=2	5.01	99.89	0.05	1.80	
Memb 3	Prim n=1	3.90	147.62	0.03	0.95	
	Prim n=2	2.95	149.98	0.02	0.71	
	Prim n=3	2.75	142.34	0.02	0.70	
Memb 4	Prim n=1	0.00	141.47	0.00	0.00	
Memb 1	Colo201 n=1	65.20	53.65	1.22	43.75	
	Colo201 n=2	52.70	58.05	0.91	32.68	
Memb 3	Colo201 n=1	288.57	98.31	2.94	105.68	
	Colo201 n=2	203.76	86.12	2.37	85.18	
	Colo201 n=3	148.93	72.36	2.06	74.10	
Memb 1	Ht29 n=1	21.06	71.01	0.30	10.68	
	Ht29 n=2	9.15	94.46	0.10	3.49	
Memb 3	Ht29 n=1	22.68	87.68	0.26	9.31	
	Ht29 n=2	75.66	103.43	0.73	26.34	
	Ht29 n=3	53.05	102.49	0.52	18.64	
Memb 1	LS174t n=1	64.56	62.34	1.04	37.29	
	LS174t n=2	54.74	72.57	0.75	27.16	
Memb 3	LS174t n=1	118.92	105.05	1.13	40.76	
	LS174t n=2	163.01	97.16	1.68	60.41	
	LS174t n=3	172.36	101.06	1.71	61.41	
Memb 1	SW480 n=1	56.70	66.50	0.85	30.70	
	SW480 n=2	26.93	89.44	0.30	10.84	
Memb 4	SW480 n=1	11.44	67.00	0.17	6.15	
	SW480 n=2	40.26	78.84	0.51	18.38	
	SW480 n=3	80.05	75.18	1.06	38.34	
			**FAR2 50 kDa**			
	**Sample**	**FAR2**	**β-actin**	**FAR2/β-actin**	**Normalization to primary cells**	**Primary cells FAR2/β-actin ratio**
Memb 2	Prim n=1	3.80	105.71	0.04	0.87	0.04
	Prim n=2	5.91	99.89	0.06	1.43	
Memb 3	Prim n=1	7.79	147.62	0.05	1.27	
	Prim n=2	8.06	149.98	0.05	1.30	
	Prim n=3	2.81	142.34	0.02	0.48	
Memb 4	Prim n=1	3.86	141.47	0.03	0.66	
Memb 2	Colo201 n=1	28.05	53.65	0.52	12.62	

	Colo201 n=2	29.25	58.05	0.50	12.16	
Memb 3	Colo201 n=1	46.47	98.31	0.47	11.41	
	Colo201 n=2	29.89	86.12	0.35	8.37	
	Colo201 n=3	22.06	72.36	0.30	7.36	
Memb 2	Ht29 n=1	42.18	71.01	0.59	14.33	
	Ht29 n=2	27.05	94.46	0.29	6.91	
Memb 3	Ht29 n=1	33.20	87.68	0.38	9.14	
	Ht29 n=2	66.59	103.43	0.64	15.53	
	Ht29 n=3	51.51	102.49	0.50	12.13	
Memb 2	LS174t n=1	20.84	62.34	0.33	8.07	
	LS174t n=2	13.70	72.57	0.19	4.56	
Memb 3	LS174t n=1	21.13	105.05	0.20	4.85	
	LS174t n=2	22.90	97.16	0.24	5.69	
	LS174t n=3	16.01	101.06	0.16	3.82	
Memb 2	SW480 n=1	42.92	66.50	0.65	15.57	
	SW480 n=2	44.80	89.44	0.50	12.09	
Memb 4	SW480 n=1	28.64	67.00	0.43	10.31	
	SW480 n=2	23.40	78.84	0.30	7.16	
	SW480 n=3	43.01	75.18	0.57	13.81	
**GNPAT 70-75 kDa**						
	**Sample**	**GNPAT**	**β-actin**	**GNPAT/β-actin**	**Normalization to primary cells**	**Primary cells GNPAT/β-actin ratio**
Memb 2	Prim n=1	22.49	105.71	0.21	1.22	0.17
	Prim n=2	28.34	99.89	0.28	1.63	
Memb 3	Prim n=1	16.25	147.62	0.11	0.63	
	Prim n=2	20.34	149.98	0.14	0.78	
	Prim n=3	13.88	142.34	0.10	0.56	
Memb 4	Prim n=1	29.16	141.47	0.21	1.18	
Memb 2	Colo201 n=1	9.20	53.65	0.17	0.98	
	Colo201 n=2	18.88	58.05	0.33	1.87	
Memb 3	Colo201 n=1	0.40	98.31	0.00	0.02	
	Colo201 n=2	0.00	86.12	0.00	0.00	
	Colo201 n=3	2.95	72.36	0.04	0.23	
Memb 2	Ht29 n=1	4.91	71.01	0.07	0.40	
	Ht29 n=2	3.78	94.46	0.04	0.23	

Memb 3	Ht29 n=1	9.52	87.68	0.11	0.62
	Ht29 n=2	11.29	103.43	0.11	0.63
	Ht29 n=3	9.41	102.49	0.09	0.53
Memb 2	LS174t n=1	47.30	62.34	0.76	4.35
	LS174t n=2	20.84	72.57	0.29	1.65
Memb 3	LS174t n=1	18.75	105.05	0.18	1.02
	LS174t n=2	43.15	97.16	0.44	2.55
	LS174t n=3	52.85	101.06	0.52	3.00
Memb 2	SW480 n=1	49.14	66.50	0.74	4.24
	SW480 n=2	63.39	89.44	0.71	4.07
Memb 4	SW480 n=1	24.67	67.00	0.37	2.11
	SW480 n=2	27.33	78.84	0.35	1.99
	SW480 n=3	30.07	75.18	0.40	2.30

Table S5. Statistical comparison of protein expression at the protein and gene level.

Western Blot Analysis										
	Prim		Colo 201		HT29		LS174t		SW480	
	Mean	±SD	Mean	±SD	Mean	±SD	Mean	±SD	Mean	±SD
FAR1	**1.0**	±0.2	**8.4**	±0.9^{aaa,b,c}	**12.4**	±1.1^{aaa}	**4.8**	±0.5^{aa,bbb}	**8.4**	±0.8^{aaa,b,c}
FAR2	**1.0**	±0.2	**10.4**	±1.1^{aaa,c}	**11.6**	±1.6^{aaa}	**5.4**	±0.7^{bb}	**11.8**^{c}	±1.5^{aaa,cc}
AGPS	**1.0**	±0.3	**68.3**	±13.4^{aaa,bbb,ddd}	**13.7**	±4.0	**45.4**	±6.7^{aa}	**20.9**	±6.0
GNPAT	**1.0**	±0.2	**0.6**	±0.4^{c,dd}	**0.5**	±0.1	**2.5**	±0.6^{b}	**2.9**	±0.5^{a,bb}

Real time PCR analysis										
	Prim		Colo 201		HT29		LS174t		SW480	
	Mean	±SD	Mean	±SD	Mean	±SD	Mean	±SD	Mean	±SD
FAR1	**1.0**	±0.0	**5.6**	±0.2^{aaa,cc,dd}	**5.5**	±0.3^{aaa}	**4.9**	±0.2^{aaa,b}	**6.3**	±0.3^{aaa,bb,ccc}
FAR2	**1.0**	±0.0	**0.3**	±0.0^{aaa,bbb,ccc}	**2.1**	±0.1^{aaa}	**7.0**	±0.2^{aaa,bbb}	**0.3**	±0.0^{aaa,bbb,ccc}
AGPS	**1.0**	±0.0	**1.3**	±0.0^{aaa,bb,ccc}	**1.5**	±0.1^{aaa}	**4.0**	±0.1^{aaa,bbb}	**1.4**	±0.1^{aaa,ccc}
GNPAT	**1.0**	±0.0	**0.9**	±0.0^{aa,bbb,ccc}	**1.2**	±0.1^{aa}	**3.6**	±0.1^{aaa,bbb}	**0.9**	±0.0^{a,bbb,ccc}

Statistical significance was assessed using ANOVA followed by Bonferroni post-test analysis. a $p < 0.05$, aa $p < 0.01$, aaa $p < 0.001$, Primary vs HT29; b $p < 0.05$, bb $p < 0.01$, bbb $p < 0.001$, Primary vs LS174t; c $p < 0.05$, cc $p < 0.01$, ccc $p < 0.001$, Primary vs SW480; d $p < 0.05$, dd $p < 0.01$, ddd $p < 0.001$, Primary vs Colo 201. Numbers in bold are the mean values.

Table S6. Membrane lipid composition of EV isolated from commercial colon cell lines.

PL or SL Class	Prim		HT29		LS174t		SW480		Colo 201	
	Mean	± SD	Mean	±SD	Mean	±SD	Mean	±SD	Mean	±SD
**PC**	**29.8**	±8.0	**61.6**	±1.5^{aa}	**58.4**	±4.1^{aa}	**60.3**	±5.8^{aa}	**48.6**	±9.6^{a}
**SM**	**34.8**	±5.5	**34.0**	±2.7	**35.4**	±2.1	**34.0**	±1.8	**28.4**	±2.8
**Cer**	**3.4**	±0.5	**1.0**	±0.3^{aaa}	**1.3**	±0.6^{aaa}	**0.8**	±0.4^{aaa}	**0.6**	±0.2^{aaa}
**PE**	**10.3**	±2.5	**0.9**	±0.4^{aaa}	**1.3**	±0.6^{aaa}	**1.3**	±0.9^{aaa}	**3.3**	±1.3^{aaa}
**PE P-**	**6.3**	±1.2	**0.9**	±0.7	**1.1**	±0.9	**1.2**	±1.7	**7.9**	±5.9
**PI**	**4.1**	±1.8	**0.4**	±0.2^{aa}	**0.6**	±0.2^{aa}	**1.1**	±0.9^{a}	**0.9**	±0.4^{aa}
**PS**	**11.3**	±4.7	**1.4**	±0.3^{a}	**1.9**	±0.7	**1.3**	±1.0^{a}	**10.3**	±5.2

Lipid classes analysis of EVs isolated from the supernatant of Primary, HT29, LS174t, SW4380 and Colo 201 cells. Statistical significance was assessed using ANOVA followed by Bonferroni post-test analysis. Statistical significance was assessed using ANOVA followed by Bonferroni post-test analysis. a $p < 0.05$, aa $p < 0.01$, aaa $p < 0.001$, Primary vs HT29, LS174t, SW480 or Colo 201; b $p < 0.05$, bb $p < 0.01$, bbb $p < 0.001$, HT29 vs LS174t, SW480 or Colo 201; c $p < 0.05$, cc $p < 0.01$, ccc $p < 0.001$, LS174t vs SW480 or Colo 201; d $p < 0.05$, dd $p < 0.01$, ddd $p < 0.001$, SW480 vs Colo 201. Abbreviations: Cer: ceramide; PC: phosphatidylcholine; PE: phosphatidylethanolamine, PE-P: alkenyl phosphatidylethanolamine; PI: phosphatidylinositol; PL: phospholipid; PS: phosphatidylserine; SM: sphingomyelin; SL: sphingolipid. Numbers in bold are the mean.

Table S7. Phospholipid and sphingolipid molecular species composition of EV isolated from commercial colon cell lines.

Molecular Species	Prim		HT29		LS174t		SW480		Colo201	
Phosphatidylcholine										
	Mean	±SD	Mean	±SD	Mean	SD	Mean	SD	Mean	SD
32:0	**12.6**	±5.5	**5.6**	±0.1	**5.7**	±0.6	**5.3**	±1.7^{a}	**7.1**	±2.2
34:2	**12.0**	±1.3	**2.2**	±0.0^{aaa}	**2.5**	±0.1^{aaa}	**2.5**	±0.3^{aaa}	**2.8**	±0.7^{aaa}

34:1	**30.5**	±6.1	**28.3**	±0.6	**28.5**	±0.9	**28.4**	±3.3	**34.7**	±4.3
36:4	**1.2**	±0.3	**1.9**	±0.1[a]	**2.0**	±0.0[aa]	**2.0**	±0.2[aa]	**1.7**	±0.3
36:3	**3.6**	±0.8	**2.9**	±0.2	**2.9**	±0.1	**3.1**	±0.2	**2.7**	±0.4
36:2	**19.9**	±5.8	**9.4**	±1.9[aa]	**8.3**	±0.3[aa]	**7.8**	±0.3[aa]	**9.6**	±1.9[aa]
36:1	**11.8**	±1.7	**17.5**	±0.8[aaa]	**17.5**	±0.4[aaa]	**16.8**	±0.3[aaa]	**14.8**	±1.0[a,b,c]
38:6	**0.2**	±0.0	**2.7**	±0.1[aaa]	**2.7**	±0.1[aaa]	**2.9**	±0.4[aaa]	**2.4**	±0.6[aaa]
38:5	**0.6**	±0.1	**5.2**	±0.2[aaa]	**5.1**	±0.2[aaa]	**5.4**	±0.8[aaa]	**4.6**	±1.0[aaa]
38:4	**3.1**	±0.7	**6.7**	±0.2[aaa]	**6.9**	±0.3[aaa]	**7.0**	±0.9[aaa]	**5.4**	±1.1[a]
38:3	**3.0**	±2.3	**6.0**	±0.3	**6.2**	±0.5	**6.4**	±1.1[a]	**4.9**	±1.1
40:7	**0.0**	±0.0	**0.6**	±0.1[aaa]	**0.6**	±0.0[aaa]	**0.7**	±0.1[aaa]	**0.5**	±0.1[aaa]
40:6	**0.2**	±0.0	**5.3**	±0.4[aaa]	**5.0**	±0.3[aaa]	**5.7**	±1.1[aaa]	**4.1**	±1.0[aaa]
40:5	**0.5**	±0.1	**5.2**	±0.1[aaa]	**5.4**	±0.3[aaa]	**5.5**	±0.9[aaa]	**4.2**	±0.8[aaa]
40:4	**0.6**	±0.2	**0.6**	±0.1	**0.5**	±0.0	**0.6**	±0.2	**0.5**	±0.1
**Phosphatidylethanolamine**										
32:0	**0.4**	±0.1	**18.3**	±11.5	**8.5**	±7.3	**10.0**	±7.1	**3.3**	±3.6
34:2	**11.6**	±3.8	**9.3**	±9.9	**7.7**	±2.7	**8.3**	±8.8	**2.0**	±0.6
34:1	**10.5**	±0.8	**30.3**	±5.3[aa]	**37.1**	±1.6[aaa]	**29.8**	±7.0[aa]	**24.1**	±6.0[a,c]
36:2	**37.5**	±2.9	**24.8**	±8.1	**24.0**	±6.2	**26.2**	±5.8	**42.4**	±5.2
36:1	**18.5**	±0.4	**23.7**	±5.2	**15.7**	±3.8	**21.0**	±2.5	**21.9**	±5.0
38:6	**0.5**	±0.3	**3.5**	±3.1	**0.5**	±0.9	**0.5**	±0.8	**0.5**	±0.8
38:5	**3.5**	±0.5	**2.2**	±1.9	**1.2**	±2.0	**1.0**	±1.7	**1.8**	±0.8
38:4	**8.0**	±0.9	**5.6**	±1.5	**3.3**	±1.7[aaa]	**3.3**	±2.3	**3.1**	±0.9[a]
38:3	**9.4**	±1.6	**0.5**	±0.9[aaa]	**2.0**	±2.3[aaa]	**0.0**	±0.0[aaa]	**0.9**	±0.4[aaa]
**PE plasmalogens**										
p16:0/20:4	**42.6**	±3.1	**38.4**	±34.1	**25.8**	±22.5	**51.9**	±6.2	**36.5**	±12.7
p16:0/22:6	**6.4**	±3.5	**28.7**	±18.5	**30.6**	±26.8	**31.0**	±17.3	**31.3**	±16.7
p18:0/20:4	**50.9**	±4.7	**32.9**	±44.4	**43.7**	±19.1	**17.2**	±11.1	**32.2**	±7.2
**Phosphatidylserine**										
34:2	**1.7**	±0.5	**0.5**	±0.1[aa]	**0.1**	±0.1[aaa]	**0.1**	±0.1[aaa]	**0.3**	±0.2[aaa]
34:1	**9.1**	±0.8	**15.7**	±3.5	**7.2**	±2.5[b]	**7.2**	±4.5[b]	**7.9**	±1.1[b]
36:3	**1.3**	±0.5	**0.4**	±0.6	**0.0**[aa]	±0.1	**0.1**	±0.1[a]	**0.3**	±0.2[a]
36:2	**16.0**	±1.9	**16.3**	±1.5	**6.5**	±2.0	**9.2**	±2.6[aa,bb]	**10.8**	±0.5[a,b]
36:1	**52.6**	±3.6	**54.6**	±5.1	**65.1**	±3.0	**66.1**	±0.4 [aa,bb]	**65.4**	±2.3 [aa,bb]
38:5	**0.4**	±0.1	**0.5**	±0.3	**0.1**	±0.1	**0.2**	±0.2	**0.1**	±0.1
38:4	**3.2**	±0.2	**5.1**	±2.0	**2.1**	±0.7	**3.6**	±0.8	**1.9**	±0.3 [bb]
38:3	**8.5**	±1.1	**3.9**	±0.8 [a]	**4.3**	±2.7 [a]	**4.5**	±1.6	**3.8**	±0.5 [a]
40:6	**0.9**	±0.7	**1.7**	±1.3	**7.4**	±2.3[aaa,bbb]	**4.2**	±0.7 [a]	**5.0**	±0.3 [aa,b]
40:5	**1.7**	±0.1	**1.3**	±0.9	**6.8**	±2.3[a,bb]	**4.5**	±1.6	**4.0**	±1.5
40:4	**4.5**	±0.8	**0.0**	±0.1[aaa]	**0.5**	±0.3[aaa]	**0.2**	±0.2 [aaa]	**0.6**	±0.3[aaa]
**Phosphatidylinositol**										
34:1	**3.5**	±2.1	**9.9**	±8.6	**10.0**	±1.4	**11.3**	±5.1	**7.0**	±2.4
36:2	**11.9**	±5.3	**4.9**	±4.6	**1.1**	±1.2	**4.7**	±1.7	**10.7**	±9.0
36:1	**4.4**	±3.1	**40.4**[aa]	±18.8	**24.8**	±1.1	**24.5**	±3.7	**16.6**[b]	±1.7
38:4	**36.8**	±3.3	**29.9**	±12.9	**29.4**	±4.8	**29.1**	±2.5	**30.3**	±2.4
38:3	**43.5**	±8.7	**14.9**[aa]	±6.2	**34.7**	±5.7	**30.4**	±7.7	**35.4**	±9.0[b]
**Sphingomyelin**										
d16:1/18:1	**1.6**	±0.5	**4.9**	±0.1[a]	**4.9**	±0.8[aaa]	**4.7**	±0.2[aaa]	**5.3**	±0.8[aaa]

d18:1/16:0	**40.2**	±6.1	**36.8**	±0.6	**38.2**	±1.5	**36.9**	±1.4	**45.4**	±5.0
d18:0/16:0	**6.9**	±3.2	**2.6**	±0.1[a]	**3.1**	±0.2[a]	**2.6**	±0.3[a]	**2.4**	±0.2[aa]
d18:1/18:1	**1.6**	±0.7	**2.3**	±0.1	**2.3**	±0.2	**2.4**	±0.3	**1.7**	±0.3
d18:1/18:0	**7.0**	±1.2	**7.8**	±0.4	**7.8**	±0.1	**7.9**	±0.8	**5.9**	±1.3
d18:1/20:0	**1.7**	±0.0	**3.1**	±0.2[aa]	**3.0**	±0.3[aa]	**3.0**	±0.2[aa]	**2.2**	±0.6
d18:1/22:1	**2.9**	±0.4	**5.3**	±0.4[a]	**5.3**	±0.3[a]	**5.4**	±0.5[aa]	**3.4**	±1.2[b,c,d]
d18:1/22:0	**7.4**	±0.6	**10.8**	±0.4[aaa]	**10.3**	±0.4[aa]	**10.2**	±0.3[aa]	**8.3**	±1.0[bb,c,d]
d18:1/24:1	**23.2**	±9.7	**19.1**	±0.7	**18.0**	±1.1	**19.0**	±0.7	**17.4**	±1.1
d18:1/24:0	**6.6**	±0.8	**7.0**	±0.4	**6.8**	±0.2	**7.4**	±0.6	**7.4**	±0.8
d18:1/26:1	**0.6**	±0.2	**0.2**	±0.1	**0.3**	±0.1	**0.4**	±0.3	**0.5**	±0.3
d18:1/26:0	**0.1**	±0.0	**0.1**	±0.1	**0.1**	±0.0	**0.1**	±0.1	**0.1**	±0.1
**Ceramides**										
d18:1/16:0	**39.9**	±7.5	**37.4**	±10.9	**33.2**	±15.6	**30.7**	±2.5	**36.4**	±10.5
d18:1/18:0	**11.3**	±1.5	**5.1**	±5.1	**1.3**	±1.6[aa]	**1.5**	±2.1[aa]	**2.3**	±2.0[aa]
d18:1/22:0	**12.2**	±1.2	**9.1**	±5.6	**8.1**	±4.6	**12.0**	±2.1	**7.7**	±2.3
d18:1/24:1	**18.4**	±6.3	**8.6**	±7.5	**11.0**	±7.7	**14.3**	±0.8	**18.2**	±4.5
d18:1/24:0	**16.5**	±3.3	**32.1**	±15.6	**34.3**	±9.5	**32.9**	±3.3	**31.7**	±6.5
d18:1/26:0	**1.6**	±0.7	**7.7**	±3.3	**12.0**	±6.2	**8.6**	±3.9	**3.8**	±4.3

Comprehensive lipidome analysis of EVs isolated from the supernatant of Primary, HT29, LS174t, SW4380 and Colo 201 cells. Statistical significance was assessed using ANOVA followed by Bonferroni post-test analysis. Statistical significance was assessed using ANOVA followed by Bonferroni post-test analysis. a $p < 0.05$, aa $p < 0.01$, aaa $p < 0.001$, Primary vs HT29, LS174t, SW480 or Colo 201; b $p < 0.05$, bb $p < 0.01$, bbb $p < 0.001$, HT29 vs LS174t, SW480 or Colo 201; c $p < 0.05$, cc $p < 0.01$, ccc $p < 0.001$, LS174t vs SW480 or Colo 201; d $p < 0.05$, dd $p < 0.01$, ddd $p < 0.001$, SW480 vs Colo 201. Numbers in bold are the mean values.

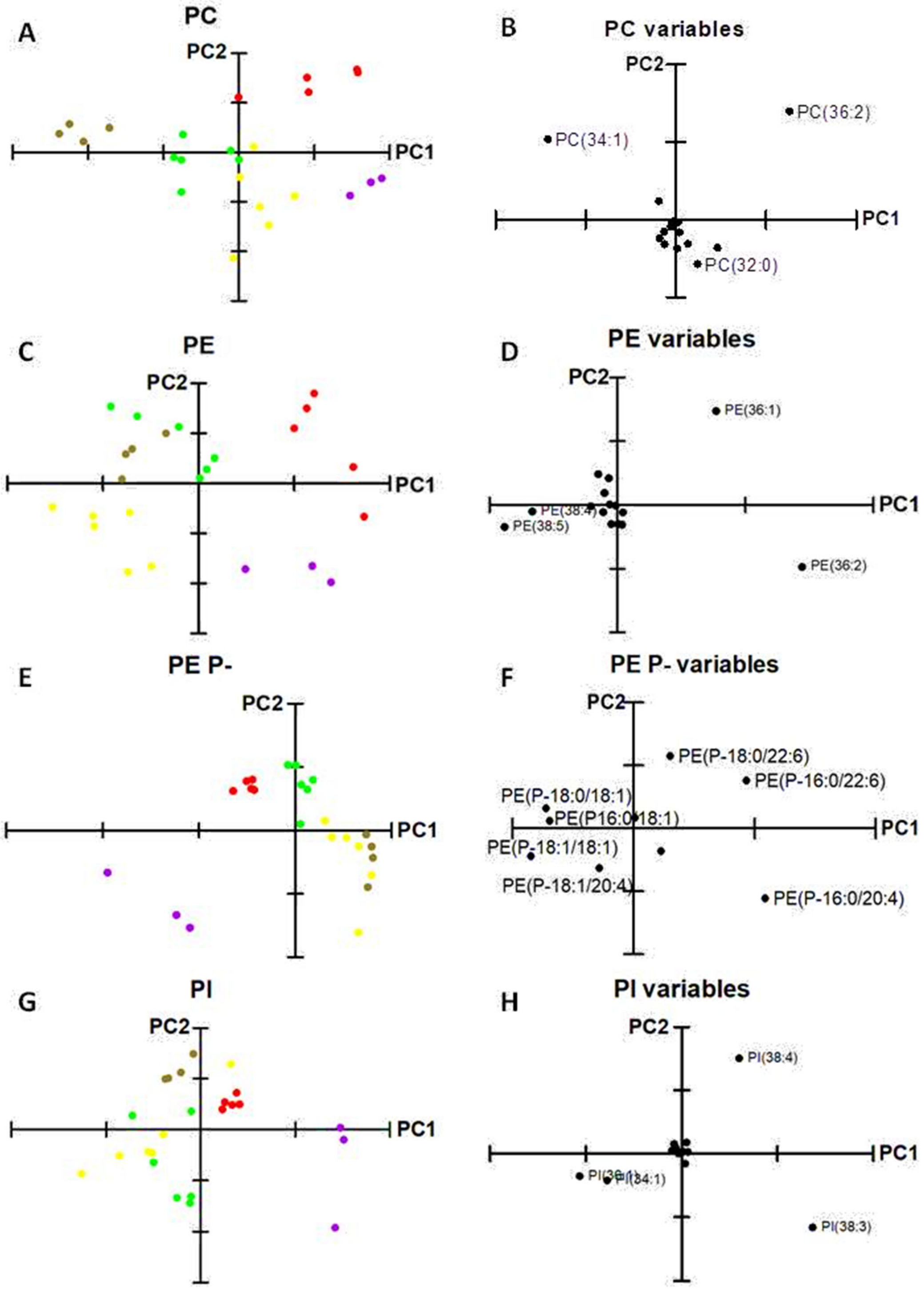
A
PC
PC2
PC1
B
PC variables
PC2
PC(36:2)
PC(34:1)
PC1
PC(32:0)
C
PE
PC2
PC1
D
PE variables
PC2
PE(36:1)
PC1
PE(38:4)
PE(38:5)
PE(36:2)
E
PE P-
PC2
PC1
F
PE P- variables
PC2
PE(P-18:0/22:6)
PE(P-16:0/22:6)
PE(P-18:0/18:1)
PE(P16:0/18:1)
PC1
PE(P-18:1/18:1)
PE(P-18:1/20:4)
PE(P-16:0/20:4)
G
PI
PC2
PC1
H
PI variables
PC2
PI(38:4)
PC1
PI(34:1)
PI(38:3)

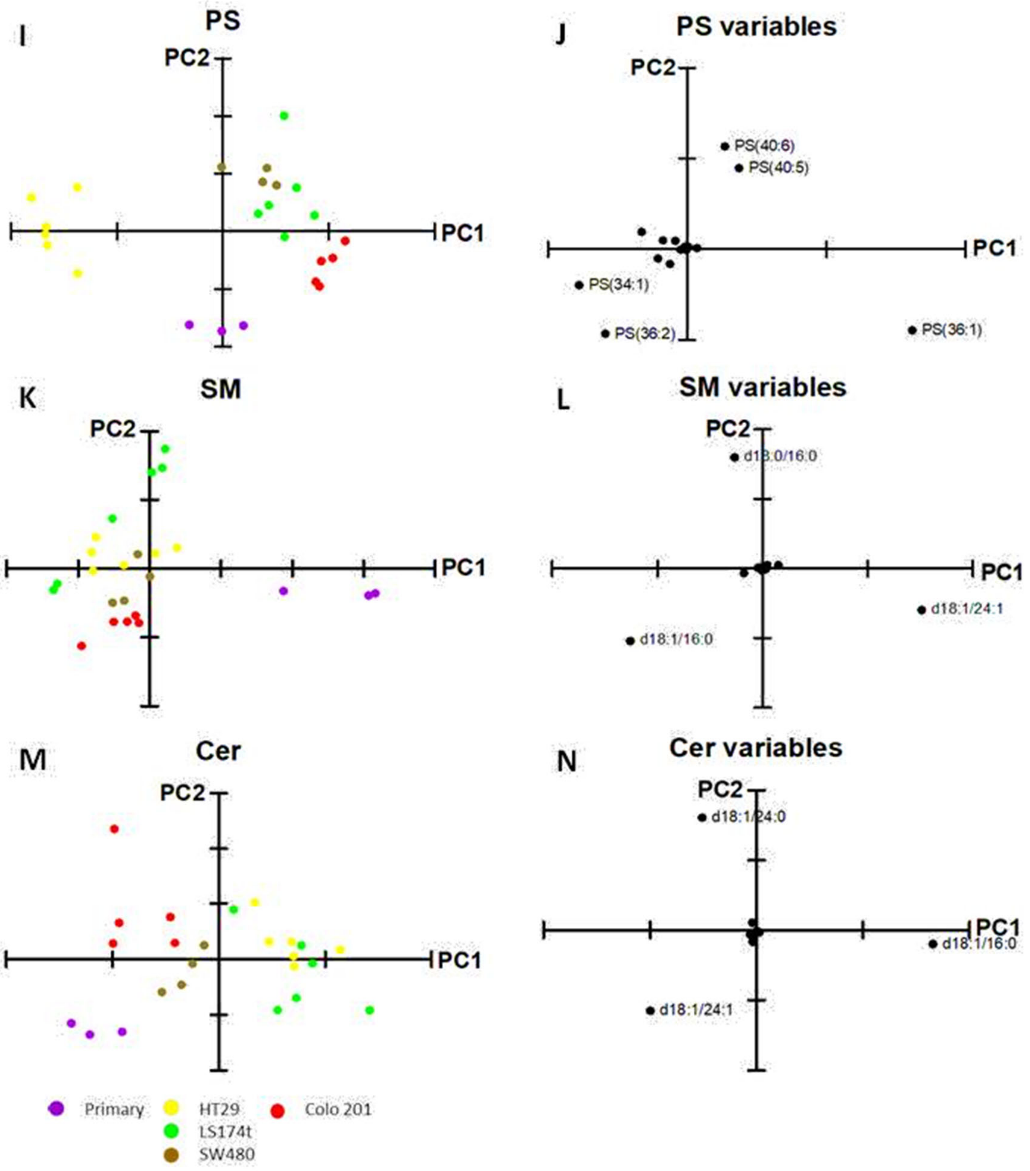

Figure S1. PCA of each membrane lipid class in colon commercial cell lines. PCA using the levels of molecular species in each membrane lipid class. Data used for the analysis were expressed as percentage of total lipid class. Explained variances: PC 82.4%, PE 65.9%, PE plasmalogens 65.9%, PI 87.5%, PS 87.5%, SM 90.6%, Cer 96.4%. Only the most influential variables are indicated in each variables graph. ● Primary, ● HT29, ● LS174t, ● SW480 and ● Colo201 cell lines.

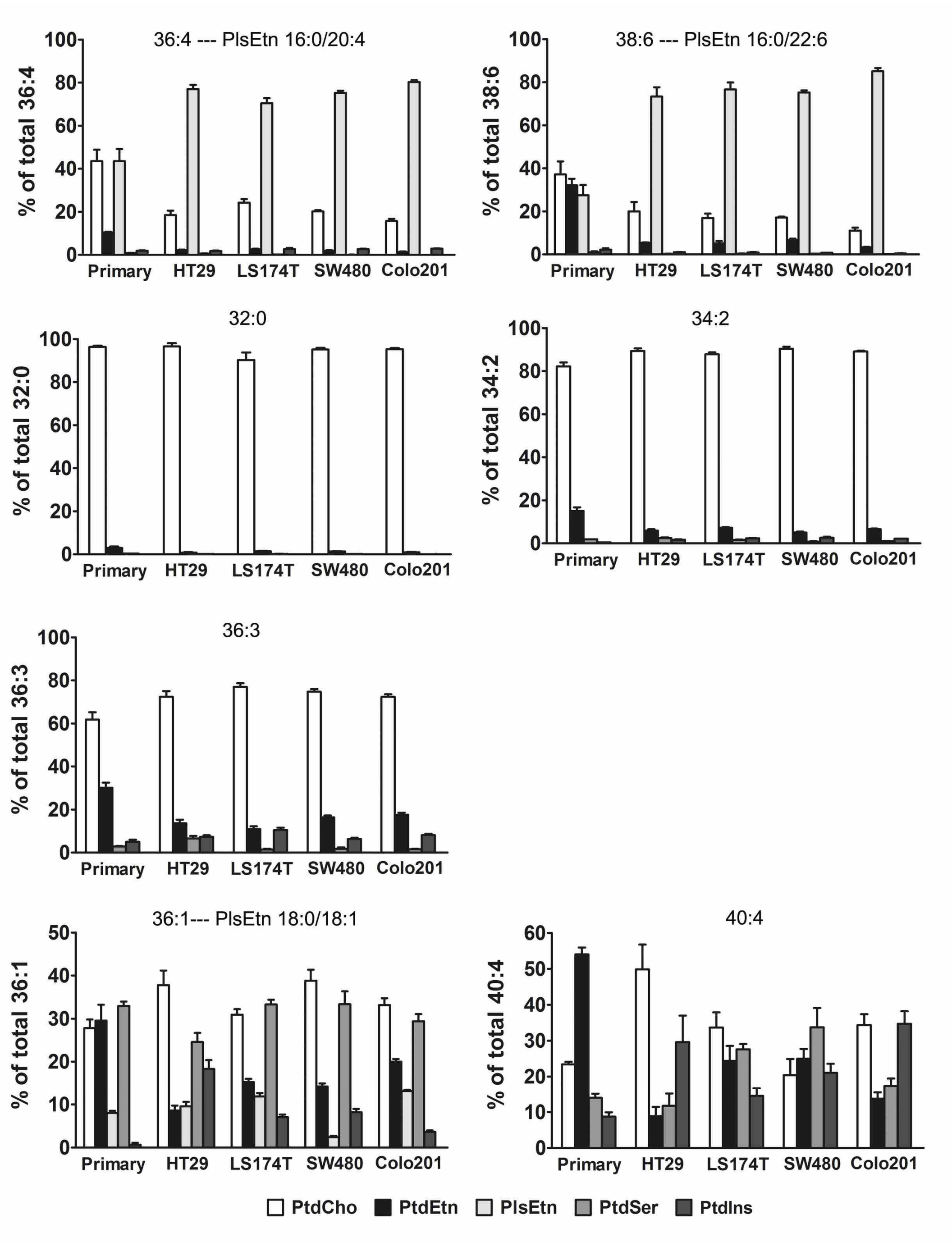

Figure S2. Distribution of the minor phospholipid specie within the main membrane phospholipid classes. Specific shift of PC and PE molecular species to sn-1 saturated /sn-2 AA or DHA - containing PE plasmalogens in cancer cells. The distribution of the total amount of particular fatty acid combination within of each membrane phospholipid class was evaluated. Values are expressed as percentage of the total amount of the selected fatty acid combination (mole %) and represent mean ± SD, n=3-6. Statistical significance was assessed using one-way ANOVA followed by Bonferroni post-test. Only significance respect primary cells are expressed. * $p < 0.05$; ** $p < 0.01$; *** $p < 0.001$. The detailed results of all the comparisons are included in Supplemental Table 3.

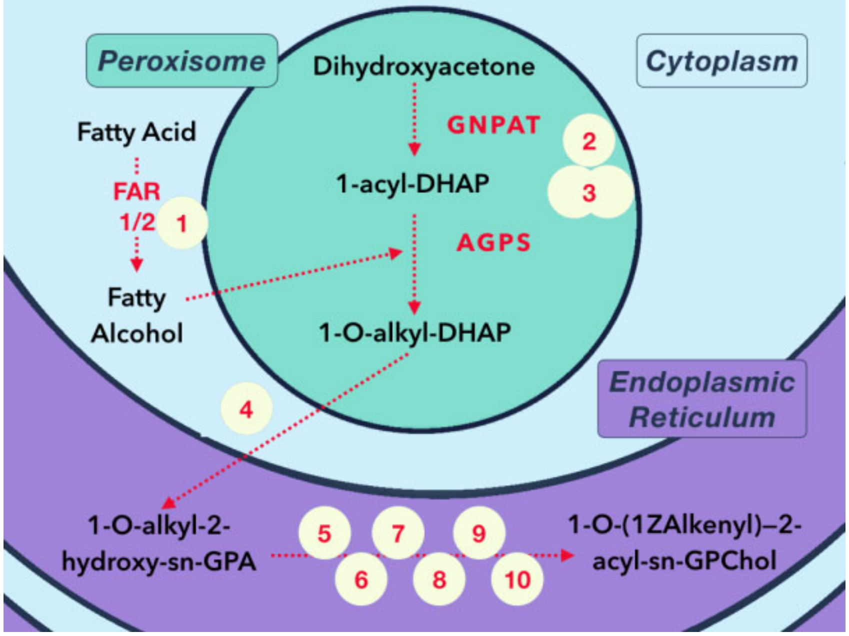

Figure S3. Scheme of the pathway for plasmalogen synthesis. Plasmalogen synthesis is a multi-step process initiated in the peroxisomes and ended in the endoplasmic reticulum. The synthesis is catalyzed by the following enzymes (1) FAR1 and 2, fatty acyl-CoA reductase 1 and 2, (2) GNPAT, glycerone phosphate O-acyltransferase (also known DHAPAT, dihydroxyacetone-phosphate acyltransferase); (3) AGPS, alkylglycerone phosphate synthase (also known ADAPS, alkyldihydroxyacetonephosphate synthase); (4) alkyl/acyl DHAP reductase, (5) alkyl/acyl glycerophosphate acyltransferase, (6) phosphatidic acid phosphatase, (7) ethanolamine (choline) phosphotransferase (8) plasmenylethanolamine desaturase, (9) phospholipase C, (10) choline phosphotransferase. Adapted from[1]. Species indicated by (Cho) represent the choline equivalent of the corresponding GPEtn species.

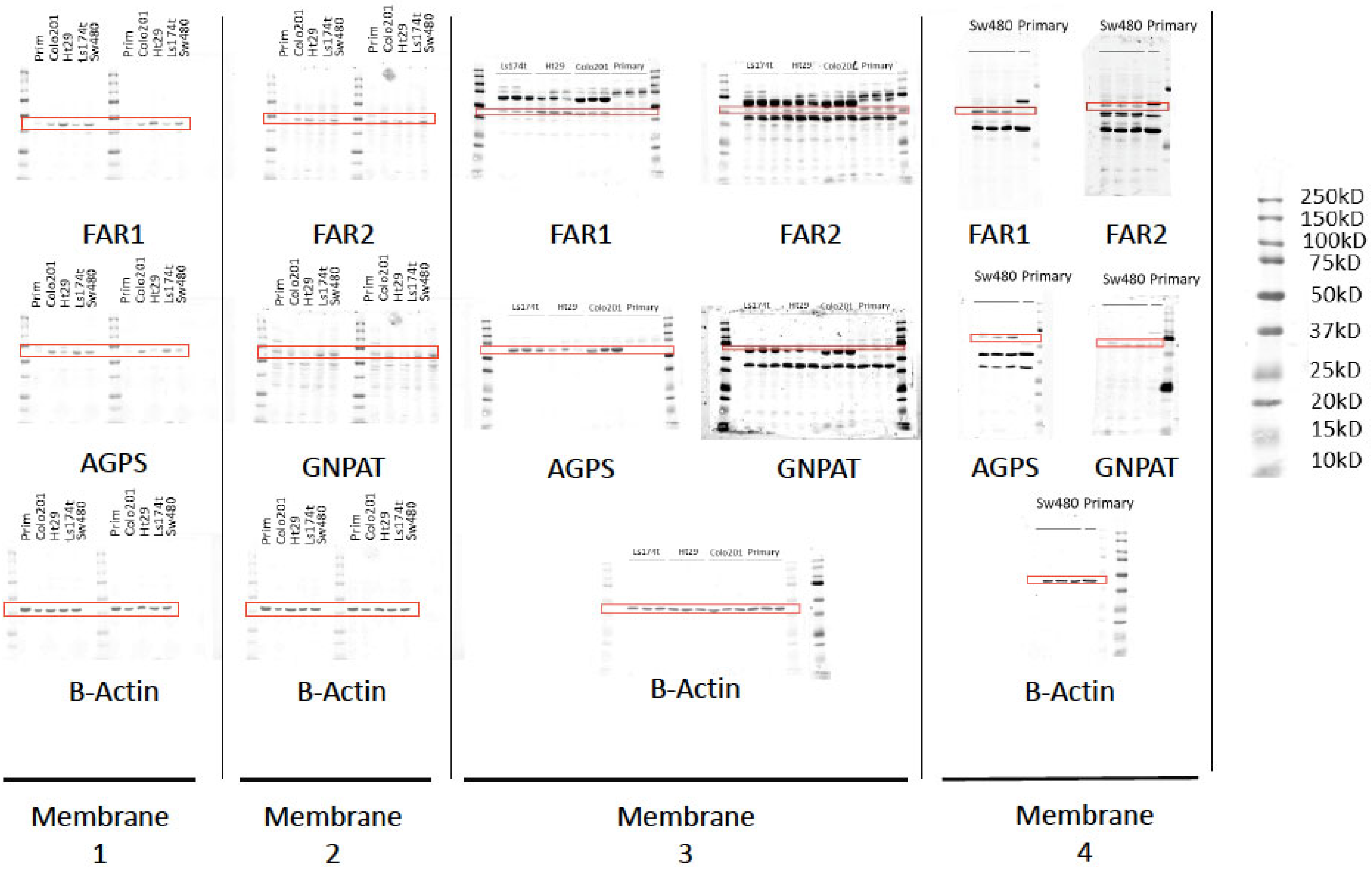

Figure S4. Original blots obtained during the study of the expression levels of plasmalogen synthetic enzymes in commercial colon cell lines.

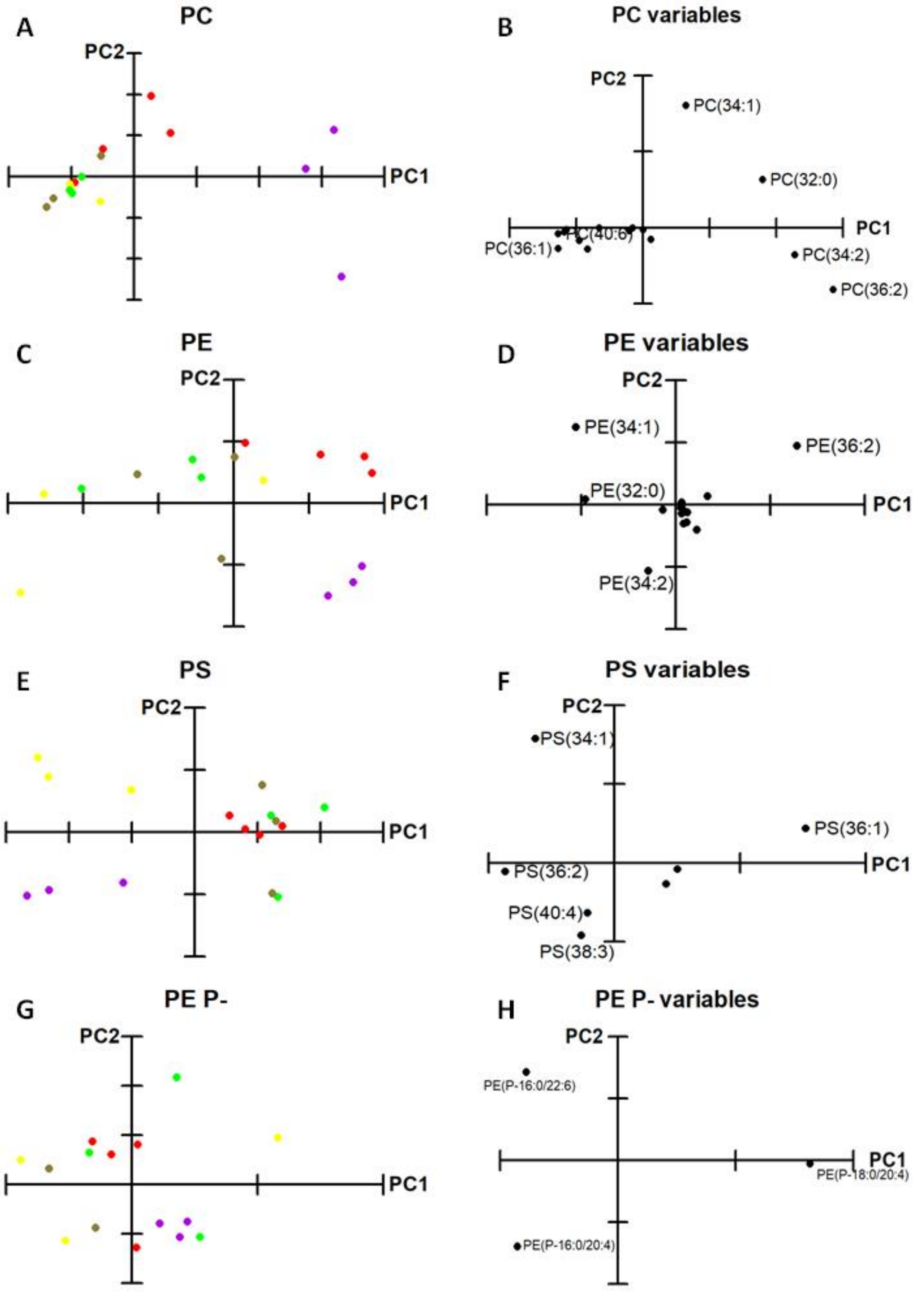

A
PC
PC2
PC1
B
PC variables
PC2
PC(34:1)
PC(32:0)
PC1
PC(40:6)
PC(36:1)
PC(34:2)
PC(36:2)
C
PE
PC2
PC1
D
PE variables
PC2
PE(34:1)
PE(36:2)
PE(32:0)
PC1
PE(34:2)
E
PS
PC2
PC1
F
PS variables
PC2
PS(34:1)
PS(36:1)
PS(36:2)
PC1
PS(40:4)
PS(38:3)
G
PE P-
PC2
PC1
H
PE P- variables
PC2
PE(P-16:0/22:6)
PC1
PE(P-18:0/20:4)
PE(P-16:0/20:4)

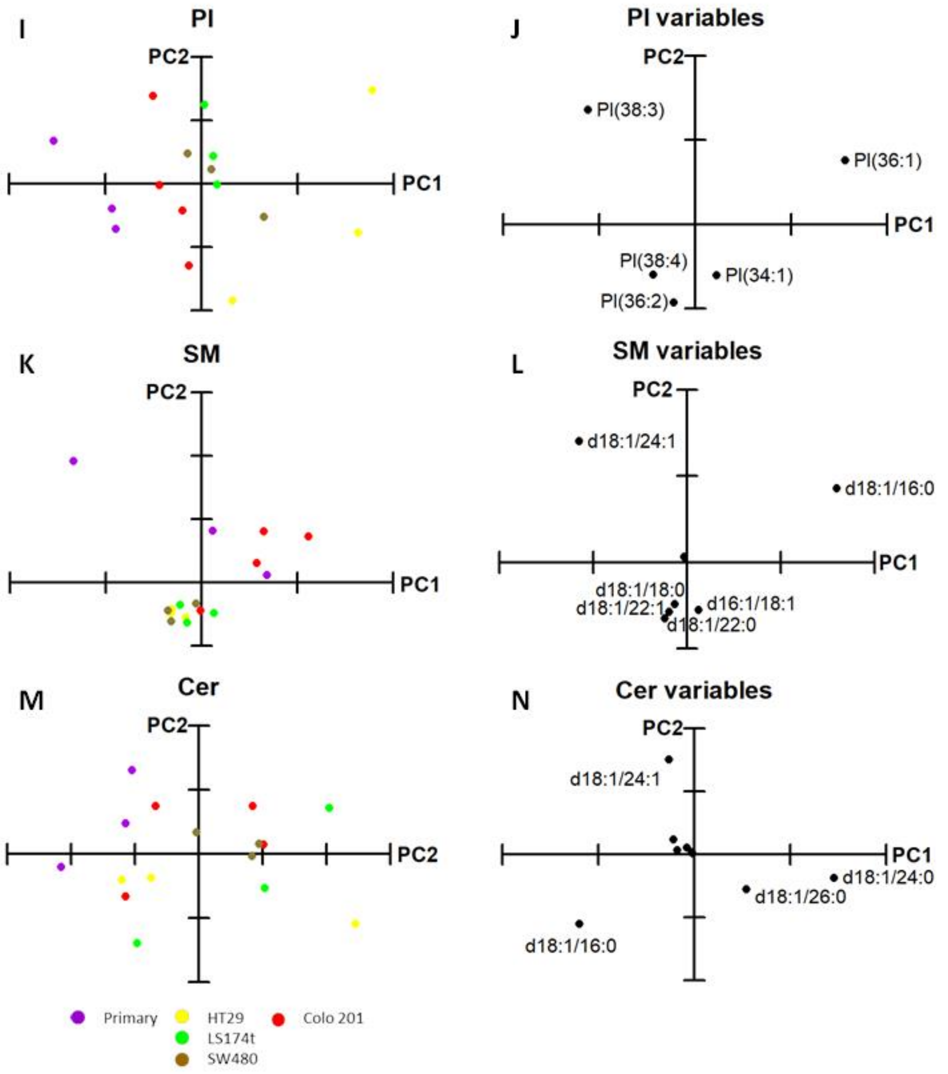

Figure S5. PCA of each membrane lipid class in EV isolated from commercial colon cell lines. PCA of lipid species analyzed in cell culture-derived EVs. Explained variances: PC 82.0%, PE 61.2%, PS 71.1%, PE plasmalogens 100%, PI 68.6%, SM 76.0%, and Cer 57.7%. For clarity, only the most influential variables are indicated at each variables PCA analysis. ● Primary, ● HT29, ● LS174t, ● SW480 and ● Colo 201 cell lines.

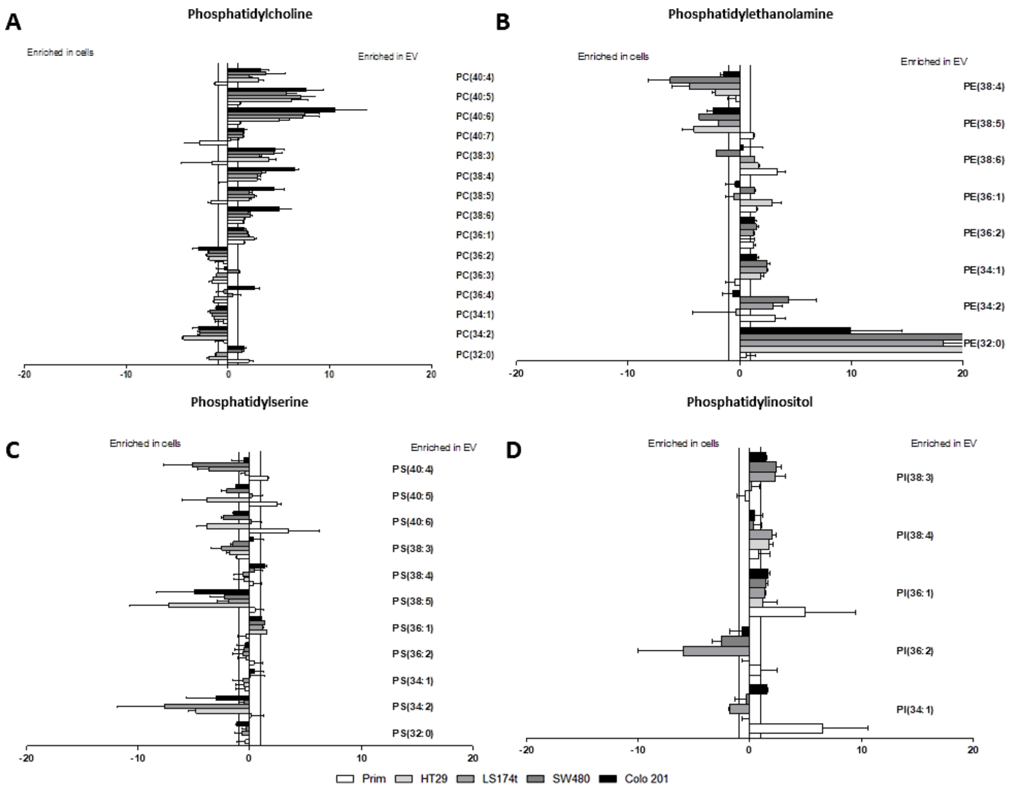

Figure S6. Membrane lipid species segregation between cells and cell-derived EV within each lipid class. Enrichment of lipid species in cells or exosomes calculated as mol% of lipids in these samples.

© 2020 by the authors. Submitted for possible open access publication under the terms and conditions of the Creative Commons Attribution (CC BY) license (http://creativecommons.org/licenses/by/4.0/).
